# Understanding premenstrual dysphoric disorder from a psychosomatic and a sensory perspective

**DOI:** 10.3389/fgwh.2025.1595083

**Published:** 2025-07-21

**Authors:** Ashita Arora, Sampurna Chakraborty, Rashmi Pandey

**Affiliations:** Amity Institute of Behavioural and Allied Sciences, Amity University, Noida, India

**Keywords:** premenstrual dysphoric disorder, interoceptive awareness, sensory processing sensitivity, emotional regulation, trauma-informed lens

## Abstract

**Aim:**

This mini-review aims to develop a multidimensional framework for Premenstrual Dysphoric Disorder (PMDD) that integrates the role of traumatic experiences, interoceptive awareness, and sensory processing sensitivity (SPS) in symptom development and maintenance.

**Background:**

PMDD is a complex disorder traditionally viewed through hormonal and mood-based lenses, but research shows that many women with PMDD experience significant emotional and physical symptoms that remain unexplained by these factors alone. Early-life trauma and interpersonal trauma may sensitize neural circuits, exacerbating symptom expression during hormonally sensitive periods.

**Method:**

A narrative synthesis of existing literature was conducted, focusing on the impact of trauma (particularly early-life and interpersonal trauma) on the hypothalamic-pituitary-adrenal (HPA) axis, sensory processing, and interoceptive awareness. The neurobiological interplay between these factors and hormonal fluctuations was examined.

**Results:**

Trauma-related dysregulation of neural circuits—including the amygdala, insula, and prefrontal cortex—heightens vulnerability to premenstrual distress by disrupting sensory and emotional processing. Heightened sensory processing and altered interoceptive awareness further amplify symptom severity during the luteal phase.

**Conclusion:**

This trauma-informed sensory framework extends current understandings of PMDD beyond hormonal and mood-based models, highlighting the importance of assessing trauma history and sensory reactivity in clinical practice. Incorporating these factors may improve diagnostic accuracy and treatment outcomes.

## Introduction: clinical features and evolving perspectives

### Background and definition

Premenstrual Dysphoric Disorder (PMDD) is a debilitating mood disorder characterized by severe emotional, cognitive, and somatic symptoms during the luteal phase of the menstrual cycle, significantly impairing daily functioning and interpersonal relationships (1). PMDD occurs after ovulation (Day 14) and persists until the onset of menstruation (Days 24–28), aligning with cyclical hormonal fluctuations (2, 3). Unlike premenstrual syndrome (PMS), which affects up to 80% of women to varying degrees, PMDD impacts approximately 3% to 8% of women of reproductive age (4). PMDD was first recognized as a distinct clinical condition in the Diagnostic and Statistical Manual of Mental Disorders, Fifth Edition (DSM-5), where it was classified under depressive disorders (5). Despite this recognition, PMDD remains absent from the International Classification of Diseases, 10th Edition (ICD-10), which limits its diagnostic use globally and contributes to its continued marginalization in clinical practice (6). Although the recently published ICD-11 includes PMDD under gynecological disorders, this placement reflects an overly biomedical view and overlooks the neuropsychiatric complexity of the condition (7).

### Symptom presentation and neurobiological perspectives

Initially, PMDD was conceptualized as an exaggerated response to hormonal fluctuations, with early research focusing on dysregulated estrogen and progesterone levels. However, subsequent studies have shown that women with PMDD do not exhibit abnormal hormone levels but rather heightened sensitivity to hormonal changes, particularly in the serotonergic and GABAergic systems (4, 8). Neuroimaging research consistently demonstrates cyclical changes in brain regions critical to emotion regulation and sensory integration, including the prefrontal cortex, amygdala, anterior insula, and anterior cingulate cortex (8–10). Recent studies also highlight the role of neuroinflammation and stress-induced alterations in the HPA axis as key contributors to PMDD pathophysiology (7, 8). These findings point to complex interactions between neurobiological processes and hormonal sensitivity that shape PMDD symptomatology.

Importantly, trauma and stress experiences can significantly affect these neural circuits. Research indicates that trauma exposure—whether in early life or adulthood—can disrupt key regions such as the amygdala, medial prefrontal cortex, and anterior insula, heightening emotional reactivity and impairing emotional regulation (11). Trauma's influence extends beyond emotional processes to interoception—the perception and interpretation of internal bodily states—which is increasingly recognized as a factor in PMDD (12, 13). For example, trauma-induced interoceptive dysregulation may cause heightened sensitivity or confusion about normal bodily sensations during the luteal phase, exacerbating symptoms of anxiety and distress (12–14).

### Risk factors and clinical implications

PMDD's development is shaped by multiple factors, including trauma (11), stress sensitivity (2), genetic predisposition (15), and sensory processing sensitivity (10). Notably, recent studies highlight that SPS—a trait characterized by heightened sensitivity to internal and external stimuli—may play a role in PMDD by amplifying emotional reactivity and sensory overwhelm (10, 16). The dynamic interplay between trauma, SPS, and interoceptive dysfunction underscores the importance of considering these factors together in understanding PMDD's heterogeneous presentation. For example, one systematic review emphasized the link between affective switching, sleep disturbances, and stress, suggesting that remote monitoring of heart rate variability, sleep, and physical activity could help identify symptom exacerbations and guide treatment interventions (17).

### Current treatment approaches and limitations

Current first-line treatments include SSRIs and hormonal therapies such as oral contraceptives (7, 18). However, recent studies reveal that many women continue to experience significant somatic and sensory symptoms, highlighting the need for more comprehensive treatment approaches (10, 18). Moreover, research on PMDD's impact on daily functioning and social behavior underscores the necessity of holistic interventions that address emotional, cognitive, and sensory domains (7, 8, 16).

### Gaps in literature and rationale for the present review

Despite growing recognition of PMDD's complexity, substantial gaps remain in our understanding. While earlier studies focused on mood symptoms and hormonal sensitivity, recent research has expanded the focus to include trauma, neuroinflammatory mechanisms, and sensory processing pathways (7, 8). For example, Henderson et al. identified a negative attentional bias in PMDD, suggesting that cognitive and behavioral factors may play a larger role in symptomatology than previously recognized. Likewise, Brown et al. emphasized the importance of real-time, remote monitoring of stress-related symptoms, highlighting the potential of digital health interventions for timely support. Importantly, intersectional factors such as gender identity, socioeconomic status, and cultural background have often been neglected in PMDD research, limiting the generalizability of findings and contributing to healthcare disparities.

Additionally, recent systematic reviews stress the need for research on affective switching and stress reactivity in PMDD, given their potential impact on suicide risk and overall well-being. The interplay between neuroendocrine sensitivity, trauma, interoceptive dysfunction, and sensory processing remains underexplored, with conflicting evidence on how these factors interact in PMDD pathophysiology (7, 17). These gaps underscore the need for integrated, interdisciplinary models that encompass both neurobiological and psychosocial dimensions.

### Aim

This paper aims to address these gaps by developing a multidimensional framework of PMDD that integrates trauma, interoceptive awareness, sensory processing sensitivity, and hormonal sensitivity. By synthesizing recent research with existing clinical knowledge, this review seeks to provide a comprehensive understanding of PMDD and inform future research directions and treatment strategies.

## Literature review and conceptual framework

### Nervous system cyclicity, trauma, and premenstrual dysphoric disorder

#### Defining trauma in this review

In this review, trauma refers to exposure to emotionally disturbing or life-threatening events that can disrupt neural systems responsible for emotion, sensory processing, and bodily awareness. While early-life trauma is often especially impactful, trauma can occur at any point in life and still influence the development and severity of PMDD symptoms (11).

#### Hormonal fluctuations and neural regulation

The menstrual cycle is influenced by the interplay of hormonal fluctuations and nervous system regulation, and it unfolds in four distinct phases—menstruation (Days 1–5), the follicular phase (Days 6–13), ovulation (Days 14–16), and the luteal phase (Days 17–28). Each phase is marked by characteristic hormonal changes that influence both physiological and psychological functioning. During menstruation, estradiol and progesterone levels are at their lowest, often contributing to increased fatigue, irritability, and heightened emotional sensitivity (2). As estradiol levels rise during the follicular phase, mood and cognitive functioning generally improve, offering a period of relative stability (3). Peak estradiol during ovulation enhances emotional resilience and cognitive flexibility, but this stabilization is short-lived as the luteal phase introduces hormonal volatility. The fluctuating progesterone and variable estradiol levels of the luteal phase contribute to emotional lability, heightened anxiety, and physical manifestations like bloating, headaches, and breast tenderness (2).

For individuals with PMDD, these normal hormonal shifts interact with an already dysregulated nervous system, significantly amplifying physical and emotional symptoms. Neuroimaging studies have demonstrated heightened amygdala activity and impaired prefrontal regulation in women with PMDD, particularly during the luteal phase, leading to intense emotional reactivity and reduced cognitive control (9). However, some studies highlight individual differences in amygdala reactivity and prefrontal modulation, suggesting that not all women with PMDD show identical neural patterns (9, 9). This points to the importance of considering individual trauma histories and neurobiological profiles.

Some studies have tried to understand the relationship between trauma and menstrual symptom dysregulation, highlighting the significant role trauma can play in exacerbating symptoms across the menstrual cycle. One study found that women with post-traumatic stress disorder (PTSD) experienced greater emotional and physical distress during the premenstrual phase compared to non-traumatized individuals (2). Another study observed that trauma survivors displayed heightened emotional reactivity and increased sensitivity to premenstrual symptoms, suggesting that trauma-related nervous system dysregulation intensifies the cyclical nature of PMDD symptoms. However, conflicting evidence exists: some studies have not found consistent differences in hormonal reactivity between trauma-exposed and non-trauma-exposed women (19). These findings underscore the need for trauma-informed perspectives when conceptualizing and treating PMDD, while also recognizing the heterogeneity in individual responses to trauma.

#### Trauma, interoceptive dysregulation, and premenstrual distress

Interoceptive awareness is the ability to perceive and interpret internal bodily signals, such as heartbeat, respiration, and visceral sensations (12). It plays a crucial role in emotion regulation, bodily self-awareness, and adaptive behavior (13). Accurate interoceptive awareness enables individuals to recognize and respond appropriately to internal physiological states, thereby supporting emotional stability. Conversely, disruptions in interoceptive processes have been linked to heightened emotional reactivity, anxiety, and emotional dysregulation (14).

Trauma profoundly affects interoceptive processing, altering key neural circuits involved in sensory and emotional integration. Neurobiological research highlights that trauma-induced hyperactivity in the amygdala and reduced connectivity in the medial prefrontal cortex (mPFC) impair the ability to differentiate between safe and threatening internal cues (11). As a result, trauma survivors frequently report heightened sensitivity to internal and external stimuli, resulting in increased vulnerability to emotional dysregulation (12).

In the context of menstrual-related symptoms, interoceptive awareness may significantly influence how individuals experience and interpret physiological changes. Hormonal fluctuations during the luteal phase can trigger physical sensations such as bloating, muscle tension, and fatigue. For individuals with heightened interoceptive sensitivity, these normal physiological changes may feel disproportionately distressing, leading to an amplification of physical symptoms and increased emotional reactivity (13). Conversely, low interoceptive accuracy can lead individuals to misinterpret benign bodily sensations as signs of serious health issues, which amplifies emotional distress and exacerbates premenstrual symptoms (2, 14). Although there are no direct studies linking interoceptive awareness to PMDD, research on menstrual symptom perception provides important insights into how interoceptive dysfunction may contribute to the emotional dysregulation, somatic complaints, and heightened anxiety commonly reported in PMDD (14). Some studies emphasize heightened anxiety sensitivity during the luteal phase, combined with misinterpretation of internal signals, as reinforcing emotional instability and physical discomfort (2). Other studies have highlighted that low interoceptive accuracy may contribute to feelings of unpredictability and confusion about bodily states, which can worsen emotional distress (13, 14). These conflicting findings suggest a complex relationship between interoception and PMDD, warranting further investigation.

Emerging research also suggests a potential connection between interoceptive awareness and sensory processing sensitivity (SPS). While interoceptive awareness focuses on internal bodily sensations, SPS involves heightened sensitivity to both internal and external sensory stimuli (10). The overlap between these two concepts could indicate a shared mechanism that contributes to emotional and somatic dysregulation in PMDD. Exploring this relationship may offer new perspectives on understanding PMDD symptomatology and improve targeted interventions.

#### Sensory processing sensitivity and its role in menstrual dysregulation

SPS is a temperament trait characterized by heightened sensitivity to both internal and external stimuli, deeper cognitive processing, and increased emotional reactivity (10). While this sensitivity can enhance adaptability in supportive environments, it may become maladaptive in contexts involving chronic stress, trauma, or hormonal fluctuations, consistent with differential susceptibility theory (10). This suggests that SPS could be an important but underexplored factor in understanding premenstrual dysregulation.

The connection between SPS and interoceptive awareness offers valuable insight into the physiological and emotional reactivity observed in individuals with premenstrual symptoms. Both phenomena involve sensitivity to internal states, and emerging evidence suggests that people with high SPS often exhibit increased interoceptive sensitivity (12). While this increased awareness can enhance self-monitoring, it can also lead to misinterpretation of normal bodily sensations, such as bloating, fatigue, or cramping during the luteal phase—thereby amplifying emotional and physical distress. In the context of PMDD, it is reasonable to hypothesize that heightened sensitivity may contribute to the amplification of symptom perception and manifestation during the luteal phase. While research does not specifically address PMDD, its findings on disrupted interoceptive processing and emotional reactivity suggest that similar mechanisms could be at play in individuals with PMDD, leading to misinterpretation of normal bodily changes as distressing or threatening (2).

Trauma may further complicate these processes by disrupting sensory processing and interoceptive regulation. Early-life trauma alters autonomic nervous system regulation, resulting in chronic hypervigilance and heightened sensory reactivity (11). Trauma survivors often report heightened sensory experiences and increased awareness of internal bodily states, which can lead to maladaptive emotional responses and heightened vulnerability to stress (11, 12). The cyclical nature of hormonal fluctuations may intensify these symptoms, particularly during the luteal phase, resulting in greater emotional and physical dysregulation. However, studies have reported conflicting results on whether heightened sensory reactivity consistently predicts PMDD severity, with some research suggesting that stress exposure and coping resources may moderate this relationship (16).

Research provides critical evidence supporting the role of sensory sensitivity in understanding premenstrual symptomatology. One study found a significantly higher prevalence of PMS and PMDD in women with autism spectrum disorder compared to the general population, attributing this to sensory hyperreactivity and emotional dysregulation (16). This finding highlights the importance of considering sensory sensitivity as a potential regulatory pathway in premenstrual symptom development. While this connection is supported in women with ASD, the broader relationship between sensory sensitivity and PMDD in the general population remains underexplored.

## Discussion: a multidimensional framework of PMDD

**Figure 1:**
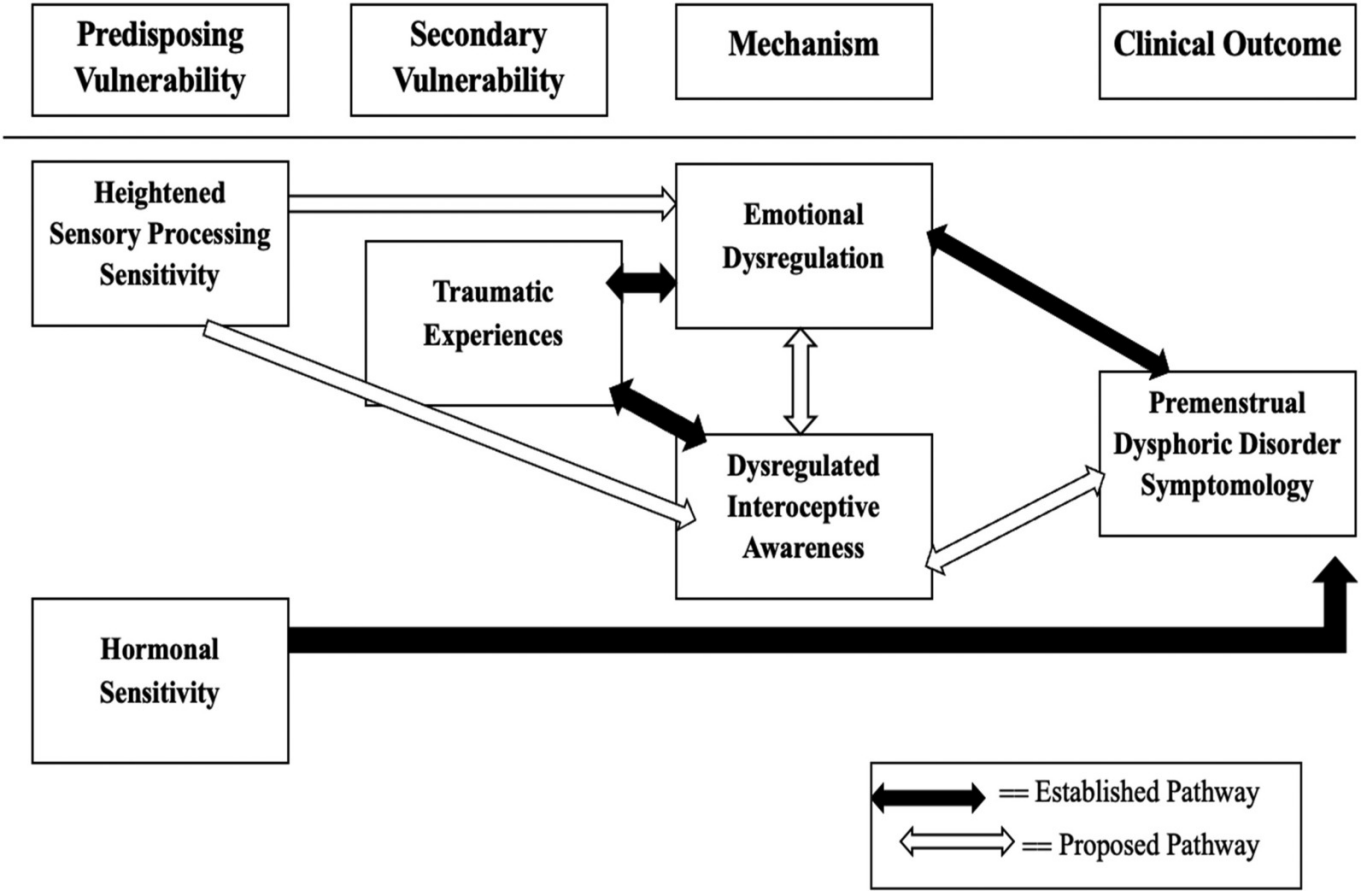
Conceptual model of the multidimensional framework for understanding premenstrual dysphoric disorder (PMDD). As depicted in Figure 1, the framework organizes contributing factors into four primary domains: Predisposing Vulnerability, Secondary Vulnerability, Mechanism, and Clinical Outcome. The black-filled arrows in the diagram represent established pathways supported by empirical research, while the white/unfilled arrows denote proposed pathways that remain to be empirically validated (20, 21). Traumatic experiences refer to exposure to emotionally disturbing or life-threatening events at any point in life that can disrupt neural systems involved in emotion regulation and bodily awareness. Hormonal sensitivity refers to heightened neural and physiological responsiveness to normal hormonal fluctuations across the menstrual cycle (Progesterone and Estrogen).

This review introduces a novel multidimensional conceptual framework (Figure 1) that integrates trauma, sensory processing sensitivity (SPS), interoceptive awareness, emotional dysregulation, and hormonal sensitivity to elucidate the complex symptomatology of Premenstrual Dysphoric Disorder (PMDD). While several of these pathways have been examined individually, this framework represents a comprehensive integration of these variables into a single model that has not yet been formally established. It is grounded in existing empirical findings while extending current theories to account for the heterogeneity in presentations of PMDD (20–24).

### Predisposing vulnerability

Within the Predisposing Vulnerability domain, SPS has been associated with heightened amygdala activation and reduced prefrontal regulatory control—neural patterns that overlap with those identified in research on PMDD (10, 25). This trait can predispose individuals to heightened emotional and sensory reactivity during hormonally sensitive phases of the menstrual cycle, potentially setting the stage for symptom development (9, 25).

### Secondary vulnerability

The Secondary Vulnerability domain encompasses traumatic experiences, which exert a significant influence on both emotional regulation and interoceptive functioning. Empirical evidence indicates that trauma can alter critical neural circuits involved in emotion and bodily awareness, such as the amygdala, medial prefrontal cortex, and anterior insula, resulting in heightened emotional reactivity and impaired interoceptive accuracy (11, 22). In addition to these neural alterations, trauma has been shown to affect hormonal sensitivity through dysregulation of the hypothalamic-pituitary-gonadal (HPG) axis, thereby increasing vulnerability to the cyclical affective disturbances characteristic of PMDD (7, 8, 20).

This dual pathway—comprising both neuroendocrine and affective mechanisms—helps explain how trauma may contribute to PMDD symptomatology even in the absence of other mediating variables. Notably, such traumatic experiences are not limited to childhood but can occur at any life stage, with enduring consequences for neurobiological systems involved in emotion regulation, stress responsivity, and hormonal integration (11). This understanding reinforces the relevance of trauma history in shaping individual differences in PMDD expression and severity.

Importantly, trauma's impact is not confined to childhood experiences; it can occur at any stage of life and still significantly shape the nervous system's responsiveness to hormonal fluctuations and stress (22, 23).

### Mechanism: emotional dysregulation and interoceptive awareness

Within the Mechanism domain, emotional dysregulation and altered interoceptive awareness are conceptualized as core mediators that link vulnerability factors (such as trauma and sensory processing sensitivity) with the clinical manifestations of PMDD. Emotional dysregulation has consistently been implicated in the pathophysiology of PMDD and is considered a defining feature of the disorder's symptomatology (7, 8, 15). This dysregulation is further exacerbated by underlying neural alterations associated with trauma exposure and high sensory processing sensitivity, particularly in regions such as the amygdala, medial prefrontal cortex, and anterior insula (10, 11, 25).

Recent evidence also points to a characteristic negative attentional bias among individuals with PMDD, marked by a heightened focus on negative stimuli and increased negative appraisal, which may further intensify affective symptoms (20). In parallel, interoceptive dysfunction can amplify both emotional and physical symptoms during the luteal phase (12–14). These interoceptive alterations are not merely secondary consequences of mood disturbances but may also stem directly from neurobiological vulnerabilities shaped by trauma and high SPS (11, 21, 25). Consequently, interoception and emotional regulation are best understood as bidirectionally linked, where impaired bodily awareness contributes to emotional instability, and vice versa.

Moreover, current conceptual models suggest that trauma and SPS may exert independent effects on interoceptive functioning, over and above their influence via emotional dysregulation. This perspective highlights the need to empirically examine these potential direct pathways, particularly through neuroimaging and psychophysiological studies that assess the interplay between sensory integration, bodily awareness, and emotion regulation (11, 21, 25).

### Clinical outcome: PMDD symptomatology

The final domain of the framework focuses on clinical outcomes, with PMDD symptomatology emerging as the result of complex, interacting mechanisms involving emotional, hormonal, and sensory processes. While the contribution of emotional dysregulation to PMDD has been well-documented and forms the basis of current diagnostic criteria (7, 8, 15), emerging models propose additional explanatory pathways that encompass sensory responsiveness and interoceptive misattunement. These processes may independently contribute to symptom severity and expression, even in the absence of overt affective disturbances (10, 20, 25).

This expanded view of PMDD emphasizes a broader symptom profile that includes not only mood-related disturbances but also somatic and sensory dimensions. For instance, individuals with elevated sensory processing sensitivity may be more reactive to hormonal shifts and environmental stimuli, which could increase vulnerability to sensory overload, fatigue, and irritability (10, 25). Similarly, interoceptive distortions may exacerbate the perception of normal luteal-phase bodily changes, reinforcing symptom severity and distress (12–14).

Hormonal sensitivity remains a central etiological factor and is understood to dynamically interact with emotional and sensory processes to modulate the onset, intensity, and variability of PMDD symptoms across cycles (7, 8). Rather than serving as an isolated driver of the disorder, hormonal fluctuations appear to act as modulators within a broader biopsychosocial network, influencing and being influenced by neurocognitive and affective mechanisms.

This reconceptualization of PMDD symptomatology supports a move away from narrowly hormone-centric models toward integrative frameworks that account for psychological and sensory vulnerabilities. It also underscores the importance of tailored clinical interventions that go beyond pharmacological symptom suppression to address trauma history, emotional processing, and interoceptive functioning.

### Integration with diagnostic criteria and treatment implications

This comprehensive framework underscores the limitations of current PMDD diagnostic criteria, which focus primarily on mood symptoms, irritability, and somatic complaints but overlook trauma, sensory processing, and interoceptive factors. By incorporating these additional dimensions, diagnostic frameworks could be refined to capture the heterogeneity of PMDD presentations better. For example, screening for SPS and interoceptive dysfunction could help identify individuals who may benefit from sensory modulation strategies or trauma-informed interventions in addition to standard treatments such as SSRIs and hormonal therapies (7, 18).

### Clinical and research implications

This integrated model bridges neuroendocrinology, affective neuroscience, and trauma psychology, offering a more nuanced and multidimensional perspective on PMDD. Future research should empirically test the proposed pathways using longitudinal and neuroimaging studies, focusing on how trauma, SPS, and interoception interact with hormonal fluctuations across the menstrual cycle. Additionally, research on neurodivergent populations and trauma survivors could elucidate how these factors shape individual differences in symptom expression and treatment responsiveness (20, 21). This approach will inform the development of personalized interventions that target both top-down and bottom-up processes, ultimately improving clinical outcomes for women with PMDD.
